# Chemical Modification of Banana Trunk Fibers for the Production of Green Composites

**DOI:** 10.3390/polym13121943

**Published:** 2021-06-11

**Authors:** Kathiresan V. Sathasivam, Mas Rosemal Hakim Mas Haris, Shivkanya Fuloria, Neeraj Kumar Fuloria, Rishabha Malviya, Vetriselvan Subramaniyan

**Affiliations:** 1 Faculty of Applied Science, AIMST University, Kedah 08100, Malaysia; 2 School of Chemical Sciences, Universiti Sains Malaysia, Minden, Penang, George Town 11800, Malaysia; rosemalharis@gmail.com; 3 Faculty of Pharmacy, AIMST University, Kedah 08100, Malaysia; shivkanya_fuloria@aimst.edu.my (S.F.); neerajkumar@aimst.edu.my (N.K.F.); 4 Department of Pharmacy, SMAS, Galgotias University, Gautam Buddh Nagar, Greater Noida 201310, India; rishabha.malviya@galgotiasuniversity.edu.in; 5 Faculty of Medicine, Bioscience and Nursing, MAHSA University, Jalan SP 2, Bandar Saujana Putra, Jenjarom Selangor, Shah Alam 42610, Malaysia; drvetriselvan@mahsa.edu.my

**Keywords:** adsorbents, adsorption, green chemistry, surface analysis, banana trunk fibers, spilled oil, hydrophobic composites

## Abstract

Natural fibers have proven to be excellent reinforcing agents in composite materials. However, a critical disadvantage of natural fibers is their hydrophilic nature. In this study, banana trunk fibers were mechanically damaged using a high-speed blender, and the resulting fibers (MDBTF) were treated with (i) stearic acid (SAMDBTF) and (ii) calcium carbonate coated with 5% (wt/wt) stearic acid (SACCMDBTF). The moisture sorption, oil sorption and thermal properties of the fibers were determined. The morphology, roughness and the functional groups present were also investigated. Study data of the present study indicate that SACCMDBTF exhibited a faster oil sorption capacity than SAMDBTF. Fast uptake of the oil occurred during the first 5 min, whereby the quantity of oil sorbed in SAMDBTF and SACCMDBTF was 5.5 and 15.0 g oil g^−1^ fiber, respectively. The results of a used engine oil uptake study revealed that SAMDBTF and SACCMDBTF sorbed 9.5 and 18.3 g/g-1 fiber, respectively, at equilibrium. The obtained results suggest that the mechanically damaged process improved the thermal stability of the fibers. This work reveals that the inclusion of stearic-acid-coated calcium carbonate into the interstices of MDBTF yields is environmentally safe for green hydrophobic composites. SACCMDBTF are used as efficient adsorbents for the removal of spilled oil on aqueous media.

## 1. Introduction

Recent decades have witnessed the extensive use of plant fibers as reinforcing agents in composites. Attributed to their low density, high mechanical qualities, wide availability and problem-free disposal, plant fibers are recognized as a viable alternative to routinely used synthetic reinforcing fibers such as carbon, glass or aramid. Fiber crops may help farmers due to their short turnaround time. The fibers produced by fiber crops are frequently quite long [1,2]. Natural fiber is a new-generation reinforcement and used as a supplement for polymer-based products made from renewable sources. Due to rising environmental concerns, the development of natural fiber composite materials or ecologically friendly composites creates importance. Natural fibers are used in various industries, including autos, furniture, packaging and construction. In comparison to synthetic fibers, natural fibers offer advantages of cheap cost, low weight, reduced damage to processing equipment, enhanced surface finish of molded component composites, good relative mechanical qualities and plentiful and renewable resources [3,4,5,6]. Furthermore, the biodegradable properties of natural fibers give less concern to environmental issues [7,8]. Fibers obtainable from cotton, kapok, kenaf, sisal, flax, palm fruit, coconut husk and banana trunk are widely used for the manufacturing of reinforced plastics, strings, cords, cables, ropes, mats, brushes, hats, baskets and fancy articles [9,10]. However, the main disadvantage of the use of these natural fibers is their susceptibility to moisture uptake [11,12]. High moisture sorption capacities of natural fibers adversely affect fiber/matrix interface interactions, resulting in material degradation that leads to the loss of strength of the manufactured composites [13]. To overcome this drawback, various means of surface modification such as esterification [14,15], alkali treatment [16], acetylation [17], peroxide treatment [18], graft copolymerization [19], silane treatment [20] and benzoylation [21] of natural fibers have been reported. There are also studies on the preparation of green composites [5,22,23,24,25]. However, the use of organic solvents, for example pyridine and dichloromethane [5] and 1,1,1-tris(*p*-hydroxyphenyl)ethane triglycidyl ether [24] for the treatment of natural fibers, may result in the inclusion of low-boiling-point organic compounds, making the resulting material not completely environmentally safe.

Fossil oil is considered the most important raw material and energy source worldwide. There are several occasions whereby oil is accidentally introduced into the environment during the production, transportation and refining process. This causes severe adverse effects on sea life and human economic activities [26,27]. Furthermore, oil spill causes strong odor, and the excessive growth of green algae may alter the color of the sea and landscape.

Banana plants belonging to the Musaceae family are cultivated primarily for their fruit. After harvesting the fruit, the matured banana trunk fibers (BTF) are normally considered as agricultural waste and disposed of at a landfill or left to decompose slowly in a plantation field. Evidence suggests the use of BTF/polyacrylamide-grafted copolymers as sorbents for the removal of Pb(II), Cd(II) and Co(II) from aqueous solutions [28,29]. A process for using various tropical plant fibers to recover spilled oil and other related hydrocarbons from land or water has been patented [30]. Studies suggest the application of banana plant waste for the removal of methylene blue, methyl red, selected heavy metals and oil from water [31,32,33,34]. Esterified BTF/poly (vinyl alcohol) blend films have also been produced as a potential replacement for edible food packaging materials [35].

Evidence suggests that stearic acid (SA) is frequently used to reduce the polarity of calcium carbonate (CaCO_3_). The method of application of SA treatments has a significant impact on the interfacial structure and distribution of CaCO_3_ thermoplastic composites. When compared to conventional surface treatments, the “complex” surface treatment produces composites with a much lower void content and higher density. This suggests that the CaCO_3_ particles and HDPE matrix are more closely packed following the “complicated” treatment procedure than with other approaches. When compared to “dry” treated CaCO_3_ composites, “wet” and “complex” treated CaCO_3_ composites offer considerably greater heat of fusion and moisture resistance. Furthermore, composites containing “wet” and “complex” treated CaCO_3_ exhibit higher tensile strength when compared to composites including “dry” treated CaCO_3_. This is due to the enhanced stearate adsorption density caused by the “wet” and “complex” treatment methods, which improves the interfacial contact between matrix and filler. The chemical adsorption of surfactant ions at the solid/liquid interface is greater than at other interfaces [36].

A study by Xiao et al. used a fractal model for capillary flow through a single tortuous capillary with roughened surfaces in fibrous porous media. The model was validated by the imbibition height and imbibition mass of capillary rise agreeing satisfactorily. The study revealed that when relative roughness increases, the imbibition height and mass of capillary decrease. The equilibrium period in a single tortuous capillary with roughened surfaces reduces as the relative roughness increases. Furthermore, with increasing imbibition duration, the imbibition height and imbibition mass of capillary also increase [37,38].

Based on previous studies, the preparation of green hydrophobic composites comprising BTF and calcium carbonate coated with stearic acid could be a good strategy. The present study reports the preparation, moisture sorption, oil adsorption and thermal properties of BTF composites, as well as the application of BTF for the removal of spilled oil on aqueous media. This paper compares the results of the present study with those of previous studies to show the efficacy of BTF as a green composite exhibiting good oil absorption capacity in the form of stearic-acid-treated, mechanically damaged banana trunk fibers (SAMDBTF) and calcium carbonate coated with 5% (*w/w*) stearic acid MDBTF (SACCMDBTF).

## 2. Materials and Methods

### 2.1. Preparation of Mechanically Damaged Banana Trunk Fibers

The preparation of BTF involved debarking and cutting the trunk of a matured banana plant (*Musa acuminate x balbisiana Colla (ABB Group) cv ‘Pisang Awak’*) into small pieces of approximately 2 cm × 2 cm in size. Next, it was subjected to washing with boiling water for 60 min, followed by cooling to ambient temperature (27–30 °C) and high-speed circular perturbation using a high-speed blender (Warring Blender, McConnellsburg, Pennsylvania, USA) for approximately 10 min to form mechanically damaged banana trunk fibers (MDBTF). Finally, the MDBTF were dried in an oven at 90 °C to a constant weight.

### 2.2. Treatment of MDBTF with Stearic Acid

The MDBTF (15 g) were added to melted stearic acid (30 g, obtained from R & M Chemicals, Subang, Selangor, Malaysia), and the hot mixture (maintained at approximately 80 °C) was stirred mechanically for 30 min. The stearic-acid-treated MDBTF, herein referred to as SAMDBTF, were isolated and placed in an oven at 70 °C until a constant weight was obtained. 

### 2.3. Treatment of MDBTF with Calcium Carbonate Coated with 5% (w/w) Stearic Acid

The MDBTF (15.0 g) were placed in 150 mL of boiling water for 30 min, and the mixture was allowed to cool to approximately 50 °C before being blended with 7.5 g of 5% (*w*/*w*) stearic-acid-coated calcium carbonate (courtesy of a local company, Usrah Jaya Enterprise, Penang, Malaysia, Registration No.: PG0052561-X) for 5–10 min. The composites, herein referred to as SACCMDBTF, were isolated and placed in an oven at 110 °C until a constant weight was obtained. To prevent the aggregation of calcium carbonate particles in water, high temperature was maintained.

### 2.4. Moisture Sorption Study

The moisture sorption study was performed in accordance with the procedure reported by Chen et al. [39], with minor modifications. All samples (BTF, MDBTF, SAMDBTF and SACCMDBTF) were dried in an oven at 110 °C for a day and then placed in a desiccator (Sigma-Aldrich, St. Louis, Missouri, United States) maintained at a relative humidity of 0% conditioned with P_2_O_5_ for a further four days. After recording the initial weight, each sample was placed in a desiccator containing a CuSO_4_∙5H_2_O saturated solution (to condition the relative humidity at 98%). Five days later, each sample was removed and weighed. The increase in weight of each sample was calculated using the expression for weight difference given in Equation (1).

(1) $$\%\;M=\frac{W_{1}-W_{o}}{W_{o}}\times 100$$

where *W*_1_ and *W_o_* are the weight of damp and dried samples, respectively. Three samples of each type of fiber (BTF, MDBTF, SAMDBTF and SACCMDBTF) were selected for the moisture sorption assessment.

### 2.5. Theoretical Approach for Thermal Analysis

The kinetics of a reaction can be calculated using the expression given in Equation (2).

(2) $$\frac{d\alpha }{dt}=kf\left(\alpha \right)\;$$

where *k* is the rate constant and *f(α)* is the reaction model, a function depending on the actual reaction mechanism. Equation (2) expresses the rate of conversion of α at a constant temperature as a function of the reactant concentration loss and rate constant. The conversion rate α is defined using the expression given in Equation (3).

(3) $$\alpha =\frac{W_{o}-W_{t}}{W_{o}-W_{f}}\times 100$$

where *W_o_*, *W_t_* and *W_f_* are the initial weights, weight at time t and final weight, respectively. The rate constant k is deduced using the expression given by the Arrhenius equation in Equation (4).

(4) k=Ae−EaRT

where *E_a_* is the activation energy, *R* is the gas constant (8.3145 J K^−1^ mol^−1^), A is the pre-exponential factor (min^−1^) and *T* is the absolute temperature in Kelvin (K). By combining Equations (3) and (4), the following relationship is derived:

(5) dαdt=Ae−EaRT. f(α)

Introducing the heating rate into Equations (5) and (6) obtains:

(6) dαdT=(Aβ)e−EaRT. f(α)

Equations (5) and (6) are the fundamental expressions of analytical methods to calculate kinetic parameters on the basis of thermal gravimetric analysis (TGA) data. The methods for calculating kinetic parameters using TGA data are summarized in Table 1. TGA was performed using a Perkin-Elmer thermograph (Model TGA 7) with a sample weight of 10 ± 1 mg. Analyses were performed under a stream of nitrogen (60 mL/min) at six different heating rates of 5, 10, 20, 50, 75 and 100 °C/min within 50 to 650 °C. Before each run, the furnace was purged with nitrogen for 20–30 min to prevent any unwanted oxidative decomposition.

### 2.6. Morphological Analysis

The morphology of the fibers used in this study was examined by scanning electron microscopy (SEM) using a Leica Cambridge AS-360 (Leica, Ernst-Leitz-Straße, Wetzlar, Germany) operated at an accelerating voltage of 15 kV. Prior to the examination, the surface of a specimen was coated with a thin layer of gold approximately 30 nm using a Polaron SC 515 sputter coater (Quorum, Laughton, East Sussex, UK).

### 2.7. Roughness Study

Roughness of the fibers used in this study was determined using Image Metrology SPIP software version 4.0.6.0. This parameter provides vital information on the level of interfacial adhesions between fibers in the matrixes when used as reinforcement.

### 2.8. Infrared Analysis

Grinded fibers were pulverized with oven-dried KBr, pressed into pellets and then analyzed using Perkin-Elmer System 2000 FTIR (Perkin- Elmer, Waltham, MA, USA). The background was scanned prior to the analysis to eliminate the unwanted bands. The samples were scanned in the mid-IR frequency range of 4000 to 400 cm^−1^ at 16 scans. All spectra were recorded at transmittance (% T) against the wavenumber (cm^−1^).

### 2.9. Oil Uptake Study

#### 2.9.1. Preparation of Weathered Oil Contaminated Seawater (WOCS)

The weathering process depends on the type and quantity of oil used [44]. In addition, the prevailing weather and sea conditions and whether the oil remains at sea or is washed ashore contribute to the severity of the weathering process. It is impossible to stimulate the entire weathering process in a controlled condition. Therefore, we focused on evaporation, photo-oxidation, microbial biodegradation and dissolution during the preparation of WOCS. A 15 L volume of fresh seawater was filtered through Whatman filter paper (No. 1) (Whatman, Florham Park, NJ, USA), and the filtrate was mixed with 100 mL of used engine oil in a glass container. A 46.0 g amount of NaNO_3_ and 3.3 g of KH_2_PO_4_ were added to the seawater as a source of nitrogen and phosphorus for the growth of microorganisms. The cylinder was exposed to sunlight and darkness in open air for 30 days to allow photo-oxidation and evaporation. The dissolved portion under the oil layer was sampled and filtered through a Whatman filter paper (No. 1). The filtered seawater was used as a standard diluent whenever dilution of WOCS was necessary.

#### 2.9.2. Equilibrium Studies

The car engine oil (Syntium 800 SL, obtained from Petronas, Kuala Lumpur, Malaysia with 10–30 W characteristics: density at 15 °C = 0.8635 kg/L, viscosity index = 141, pour point =−33 °C and flash point = 228 °C) was mixed with 30 mL of water inside a 250 mL beaker for 30 min at 150 rpm in an orbital shaker (Cole-Parmer, East Bunker Ct, VH, USA). The amount of engine oil used was in the range of 0.4 to 3.2 g. The agitation caused the oil to form a layer on the surface. A 1.0 g amount of each sorbent (BTF or MDBTF or SAMDBTF or SACCMDBTF) was then placed onto the oil. The sorbent was removed from the beaker periodically (1, 2, 5, 10, 15, 30 and 60 min). Subsequently, excess oil was separated from the sorbent by centrifuging at 2000 rpm (higher rpm tends to drip the sorbed oil away from the sorbent) for 10 min. The oil-loaded sorbent was scraped and dried in an oven at 90 °C until a constant weight was obtained. The amount of oil sorbed was computed by subtracting the weight of the fibers before sorption from that after sorption.

#### 2.9.3. Study Reusability of Sorbents

A reusability test was performed for SACCMDBTF, as it showed the best oil sorption capacity compared to that of the others. The test was performed by taking 1.0 g of oil-loaded SACCMDBTF with a weight-to-weight ratio of oil to water of approximately 1:10. After the amount of sorbed oil was determined, the oil-loaded SACCMDBTF was squeezed manually using filter paper to extract any oil that remained, and the resulting SACCMDBTF was weighed again. The test was repeated five times.

## 3. Results and Discussion

### 3.1. Moisture Sorption Study

Moisture sorption capacity was found to increase in the order of SAMDBTF (1.1%) < SACCMDBTF (1.8%) < MDBTF (6.0%) ≤ BTF (6.1%). The moisture sorption capacities of BTF and MDBTF were practically the same, suggesting that although the mechanical damage process likely resulted in some removal of hemicelluloses, amorphous cellulose and waxy cuticle layers, the uptake of water via capillary action remained virtually unchanged. The presence of stearic acid made the fibers’ surfaces hydrophobic [45], thus preventing the accumulation of water on the surfaces of SAMDBTF and SACCMDBTF. Thermal breakdown of cellulose fibers was accomplished in a single step. The thermograms of cellulose esters revealed a two-step degradation process, which was most likely governed by crosslinking processes that occur during heat decomposition.

### 3.2. Thermal Analysis

**Figure 1:**
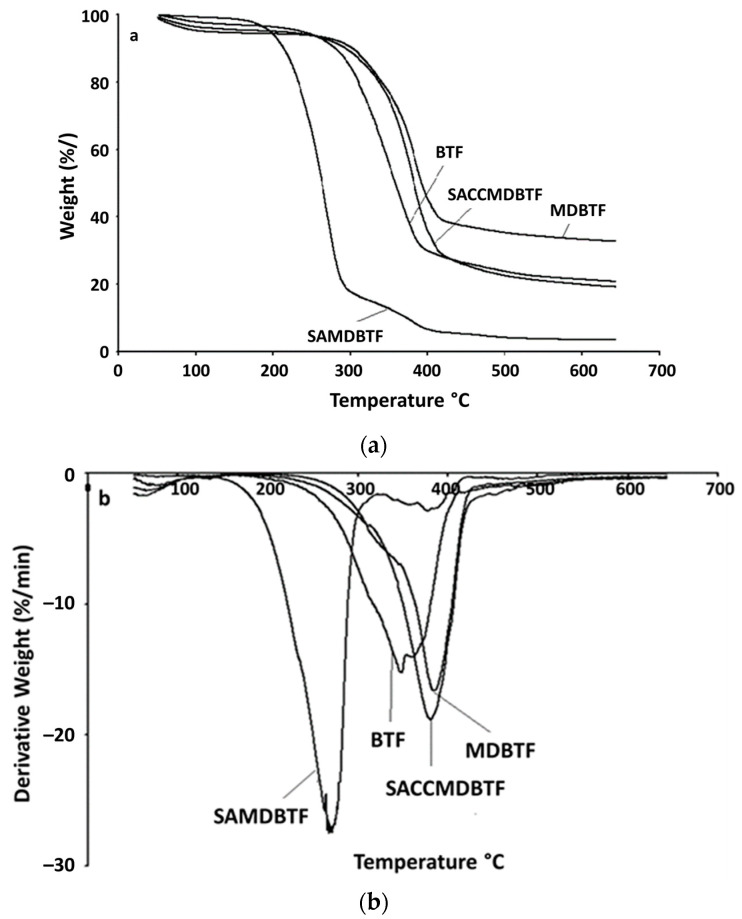
Plots of the thermogravimetric analysis: (**a**) TGA and (**b**) DTG curves of BTF, MDBTF, SACCMDBTF and SAMDBTF.

The decomposition temperature increased in the order of SAMDBTF (272.47 °C) < BTF (350.17 °C) < SACCMDBTF (382.78 °C) < MDBTF (388.43 °C). The obtained results suggest that the mechanically damaged process improved the thermal stability of the fibers, which also increased the char yield, as shown in Figure 1. Figure 1a shows that MDBTF produce more char yield as compared to BTF. The mechanically damaged process caused the fibers to lose a small amount of their chemical compositions. This process appears to remove the amorphous constituents (waxy cuticle layers and hemicelluloses) in the fibers. However, this process did not significantly remove lignin, as the char yield of the fibers increased. This can be explained further through the differential thermogravimetry (DTG) curves (Figure 1b) of the fibers. The DTG results indicate the peak position of the mechanically damaged fibers that was shifted to a higher temperature as compared to the long fibers. Results obtained show the formation of a shoulder peak at the lower temperature of the mechanically damaged fibers. The temperature of the shoulder peak was similar to the peak temperature of the long fibers. This suggests that the removed composition that degraded at the temperature of 260–280 °C was hemicelluloses, waxy cuticle layers and amorphous cellulose. These compositions were degraded by heat much before lignin [46], and thus the removal of these constituents increased the crystallinity of the fibers, which, in return, increased their thermal stability. The shifting of the peak position to a higher temperature was due to these changes. A significant decrease in decomposition temperature was observed in SAMDBTF. A similar trend was observed by Jandura et al. [47]. Stearic acid has the effect of reducing the crystallinity of the fibers. Thus, the stearic-acid-treated fibers showed a reduction in the degradation temperature. The high thermal stability of calcium carbonate and the low amount of stearic acid present in SACCMDBTF improved the thermal stability as compared to SAMDBTF.

### 3.3. Kinetics Study

The average apparent activation energies for all the treated fibers were calculated using the Kissinger equation [40] and the isoconversional Friedman [41] and Flynn–Wall–Ozawa (F–W–O) methods [42,43]. Yao et al. suggest that high conversion periods are not recommended, as the reaction mechanism might change [48]. Hence, a conversion range of 0.2–0.6 was chosen for this study. In all cases, a linear correlation greater than 0.95 among the independent and dependent variables was observed. The Kissinger method showed activation energies of 115.40 and 167.12 kJ/mol for SAMDBTF and SACCMDBTF, respectively. Similar results were obtained for the Friedman (105.95 and 170.29 kJ/mol for SAMDBTF and SACCMDBTF, respectively) and F–W–O (113.97 and 174.77 kJ/mol for SAMDBTF and SACCMDBTF, respectively) methods (Table 2). The results indicate that the surface modification reduced the activation energy of BTF. Activation energy indicates the crystallinity of fibers [49]. SAMDBTF showed a significant reduction in the activation energy compared to BTF. Hence, the modification with stearic acid reduced the crystallinity of the fibers and thus reduced the activation energy. The presence of stearic acid reduced the crystallinity of the fibers, which corresponded to a minor reduction in the activation energy despite the inclusion of calcium carbonate, a high thermally stable compound, in SACCMDBTF.

### 3.4. Morphological Analysis

**Figure 2:**
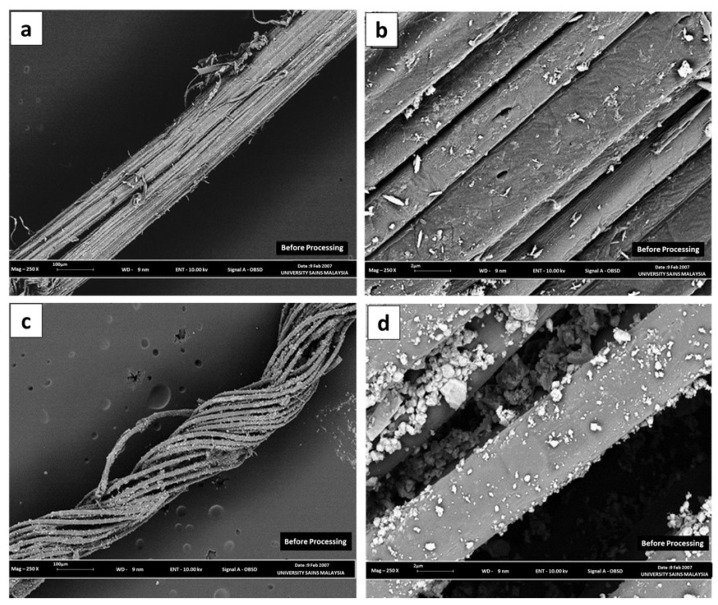
Scanning electron microscope images: (**a**) BTF at 100 μm, (**b**) BTF at 2 μm, (**c**) SACCMDBTF at 100 μm and (**d**) SACCMDBTF at 2 μm.

The morphological analysis of BTF and its composites involved SEM analysis. Figure 2 shows the SEM micrographs of BTF at 100 µm (Figure 2a), BTF at 2 µm (Figure 2b), SACCMDBTF at 100 µm (Figure 2c) and SACCMDBTF at 2 µm (Figure 2d). The micrographs of SACCMDBTF clearly indicate that cracks and voids were created due to the mechanical treatment, and stearic-acid-coated calcium carbonate was embedded in the cracks.

### 3.5. Roughness Analysis

**Figure 3:**
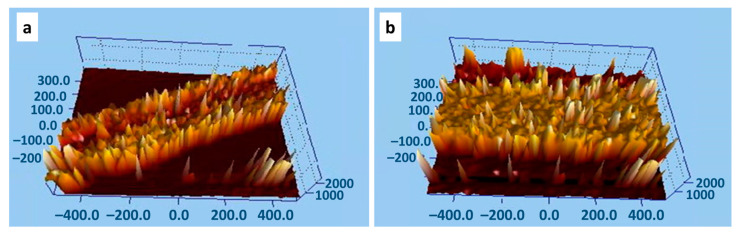
Roughness images: (**a**) BTF and (**b**) SACCMDBTF.

Figure 3 depicts the surface morphology of BTF (Figure 3a) and SACCMDBTF (Figure 3b). The parameters determined in this study are the roughness, (Sa), and mean roughness, (S mean). The Sa and Smean of the BTF and SACCMDBTF are 4.46 × 106 pm and 6.42 × 106 pm and 6.39 × 106 pm and 8.62 × 106 pm, respectively. The data show an increase in the roughness of SACCMDBTF as compared to that of BTF. This suggests that the treated fibers have better matrix adhesion and oil adsorption capacity.

### 3.6. Infrared Spectra Analysis

**Figure 4:**
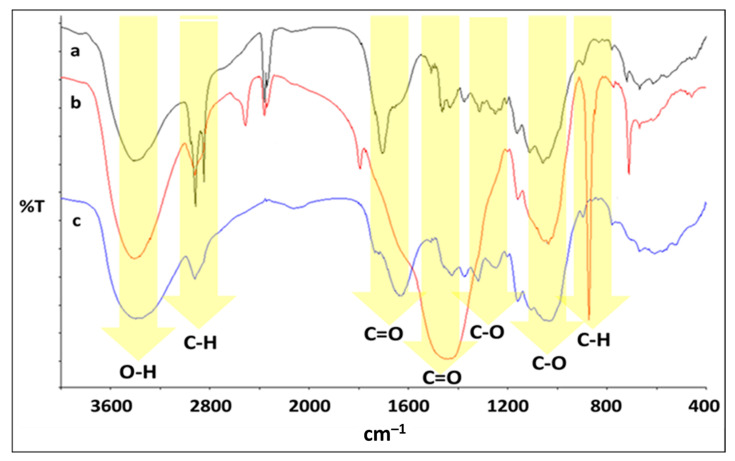
Plots of the FTIR spectrum: (a) SAMDBTF, (b) SACCMDBTF and (c) MDBTF.

The FTIR spectra of MDBTF, SAMDBTF and SACCMDBTF are shown in Figure 4. The assignments of the important absorption bands for BTF were performed as per the standard of the literature [32].

The identification of the relevant absorption bands observed in the FTIR spectra of SAMDBTF and SACCMDBTF is hereby described. Strong absorption at approximately 3400 cm^−1^ was assigned to the stretching of hydroxyl groups. The bands at 2918 and 2849 cm^−1^ were assigned to the antisymmetric and symmetric CH_2_ stretching vibrations of the stearic acid hydrocarbon chains, respectively [50]. These stretching vibrations appear more intense in SAMDBTF as compared to SACCMDBTF due to the increase in the amount of stearic acid. The bands at 1707 and 1464 cm^−1^ were assigned to the stretching modes of the C=O in the carboxylate group and scissoring vibration of CH_2_, respectively [51]. The bands at 1423, 1373 and 1318 cm^−1^ were attributed to CH_2_ bending, OH bending and C–O skeletal vibrations, respectively. The band at 1250 cm^−1^ corresponded to C–O stretching in hemicelluloses. The bands at 1162, 1110 and 1054 cm^−1^ corresponded to C–O antisymmetric bridge stretching, C–O–H stretching and C–O–C pyranose ring skeletal vibrations, respectively. As for SACCMDBTF, the presence of calcium carbonate is well defined by the presence of a very intense broad band centering at 1420 cm^−1^ and bands at 1795, 873 and 712 cm^−1^ [52,53].

### 3.7. Oil Uptake Study

**Figure 5:**
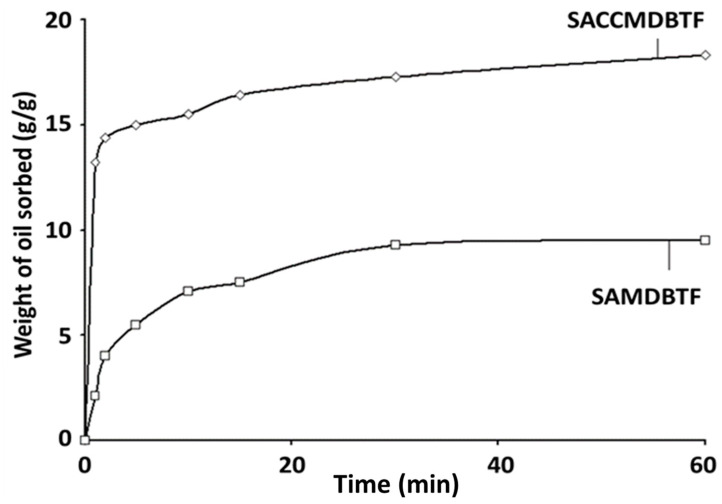
Quantity of used car engine oil sorbed (g oil g^–1^ fiber) with respect to time (min) by SACCMDBTF and SAMDBTF.

The BTF and MDBTF both sorbed the WOCS (comprising car engine oil and seawater). Consequently, these fibers were submerged in the water for 60 min. However, in the case of SAMDBTF and SACCMDBTF, due to the presence of stearic acid, the materials are hydrophobic and, as such, were found to preferentially sorb the oil and remain afloat. Figure 5 indicates that SACCMDBTF exhibited a superior oil sorption capacity than SAMDBTF. 

Fast uptake of the oil occurred during the first 5 min, whereby the quantity of oil sorbed in SAMDBTF and SACCMDBTF was 5.5 and 15.0 g oil g^−1^ fiber, respectively. The equilibrium was reached at approximately 60 min with an adsorption capacity of 9.5 and 18.3 g oil g^−1^ fiber for the former and latter, respectively. The notion that the adsorption capacity of polymeric sorbents for oil floating on water must be considerably lower than the actual or effective adsorption capacity of the materials, as pointed out by Hussein et al. [29], is a reasonable point of concern. The present work considered this issue and, hereby, addressed it accordingly. Based on the observed fast oil uptake and diminishing moisture sorbed (as revealed by the results of the moisture adsorption study described above) and also likely minimal water sorbed, if any (justifiable by the fact that the composites when placed on water remained afloat for days even when being subjected to swirling condition), the determined oil adsorption capacity for SAMDBTF and SACCMDBTF should be similar or practically equal to their respective effective oil adsorption capacity. The superior oil adsorption capacity of SACCMDBTF can be attributed due to the presence of stearic-acid-coated calcium carbonate within the interstices of the mechanically damaged fibers, which also sorbed oil readily and, therefore, led to an increase in the total amount of oil sorbed as compared to that in SAMDBTF, whereby the oil uptake occurred solely or primarily via capillary action. The calcium carbonate has up to a 13–21 g/100 g capacity of oil absorption, so it can influence the oil uptake of stearic-acid-coated CaCO_3_ [54].

### 3.8. Reusability of Sorbents

**Figure 6:**
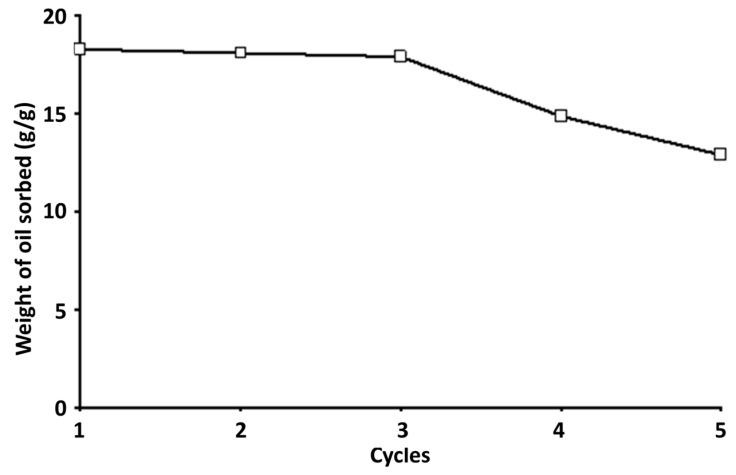
Adsorption of oil by SACCMDBTF during five repeated biosorption cycles in the orbital shaker at 150 rpm, with a contact time of 30 min, for each cycle of adsorption.

The sorbed SACCMDBTF was reused in several adsorption–desorption cycles to ascertain the efficiency of the rejuvenated biomass as an oil removal sorbent from the WOCS. The quantities of oil sorbed and desorbed from the WOCS were determined for five repeated cycles (Figure 6). One-way ANOVA, followed by Duncan’s multiple test, indicated that no statistical weight difference in the adsorption and desorption of oil on the first three successive adsorption–desorption cycles was observed. However, oil adsorption declined by 18.7% and 29.3% on Cycles 4 and 5, respectively. This result indicates that the rejuvenated SACCMDBTF was also able to retain its original efficiency to sorb oil during three repeated cycles. Matolia et al. used the one-way ANOVA, followed by Duncan’s multiple test, to determine the efficacy of the entrapment of chitosan in a fixed-down-flow column bed, which enhances the removal of triclosan from water [55].

The data of the present study indicate that SACCMDBTF exhibited a faster oil sorption capacity than SAMDBTF. Fast uptake of the oil occurred during the first 5 min, whereby the quantity of oil sorbed in SAMDBTF and SACCMDBTF was 5.5 and 15.0 g oil g^–1^ fiber, respectively. The results of a used engine oil uptake study revealed that SAMDBTF and SACCMDBTF sorbed 9.5 and 18.3 g/g^–1^ fiber, respectively, at equilibrium. The obtained results suggest that the mechanically damaged process improved the thermal stability of the fibers. This work reveals that the inclusion of stearic-acid-coated calcium carbonate into the interstices of MDBTF yields is environmentally safe or green hydrophobic composites. SACCMDBTF are used as efficient adsorbents for the removal of spilled oil on aqueous media.

## 4. Conclusions

This present work proves that the oil adsorption capacity of the produced green hydrophobic composites increased markedly as compared to that of the untreated BTF. In particular, the mechanically damaged BTF with the inclusion of 5% (wt/wt) stearic-acid-coated calcium carbonate (SACCMDBTF) were found to have an impressive oil adsorption capacity of 18.3 g oil g^−1^ fiber for the used car engine oil. Besides exhibiting a decrease in moisture sorption properties, SACCMDBTF showed improved roughness properties, suggesting an increase in fiber/matrix interface interactions. Furthermore, the reusability of SACCMDBTF was impressive; viz., the material was able to retain its original efficiency to sorb oil for at least three repeated cycles. The obtained results demonstrate that BTF, which are abundantly available but generally discarded as agricultural waste, can be easily and inexpensively converted into green hydrophobic composites and utilized as efficient sorbents for the removal of spilled oil in aqueous media. This research shows that the inclusion of stearic-acid-coated calcium carbonate to the interstices of MDBTF yield is environment friendly, and SACCMDBTF can be used as effective adsorbents for removing spilled oil from aqueous media. With this study, we could find an efficient method for oil absorption, and its influence on composites is described in detail. Finally, future growth patterns in entirely green composites are forecasted.

## Figures and Tables

**Table 1:** Methods for calculating the kinetic parameters using TGA data.

Method	Expression	Plots	Reference
Kissinger	−ln(βTm2)=EaRTm−ln(AREa)	ln(βTm2)against 1Tm	[40]
Friedman	ln(dαdt)=ln(Z)+nln(1−α)−EaRT	ln(dαdt) against 1T	[41]
Flynn–Wall–Ozawa	logβ=log(AEoRg(α))−2.315−0.4567EaRT	logβagainst 1T	[42,43]

**Table 2:** Average activation energy of SAMDBTF and SACCMDBTF calculated by the Friedman and Flynn–Wall–Ozawa methods at a period of α = 0.2–0.6.

Fibers	Conversion, α	Friedman Ea (kJ/Mol)	F–W–O Ea (kJ/Mol)
SAMBTF	0.2	104.67	112.88
	0.3	104.89	112.95
	0.4	105.59	113.52
	0.5	106.73	114.70
	0.6	107.89	115.78
	Mean ± SD	105.95 ± 1.34	113.97 ± 1.25
SACCMBTF	0.2	189.57	192.98
	0.3	172.11	176.56
	0.4	162.96	167.49
	0.5	153.88	158.39
	0.6	172.94	178.15
	Mean ± SD	170.29 ± 13.27	174.77 ± 12.94

## Data Availability

The data presented in this study are available on request from the corresponding author.
